# A non-optimal cervicovaginal microbiota in pregnancy is associated with a distinct metabolomic signature among non-Hispanic Black individuals

**DOI:** 10.1038/s41598-021-02304-0

**Published:** 2021-11-23

**Authors:** Kristin D. Gerson, Jingqiu Liao, Clare McCarthy, Heather H. Burris, Tal Korem, Maayan Levy, Jacques Ravel, Michal A. Elovitz

**Affiliations:** 1 Department of OB/GYN, Center for Research on Reproduction and Women’s Health, Perelman School of Medicine, University of Pennsylvania, Biomedical Research Building II/III, 1351, 421 Curie Blvd, Philadelphia, PA 19104-6160 USA; 2 Program for Mathematical Genomics, Department of Systems Biology, Columbia University Irving Medical Center, New York, NY 10032 USA; 3 Department of Obstetrics and Gynecology, Columbia University Irving Medical Center, New York, NY 10032 USA; 4 CIFAR Azrieli Global Scholars Program, CIFAR, Toronto, Canada; 5 Department of Microbiology, Perelman School of Medicine, University of Pennsylvania, Philadelphia, PA 19104 USA; 6 Institute for Genome Sciences, University of Maryland School of Medicine, Baltimore, MD 21201 USA

**Keywords:** Reproductive biology, Reproductive disorders

## Abstract

Biomechanical and molecular processes of premature cervical remodeling preceding spontaneous preterm birth (sPTB) likely result from interactions between the cervicovaginal microbiota and host immune responses. A non-optimal cervicovaginal microbiota confers increased risk of sPTB. The cervicovaginal space is metabolically active in pregancy; microbiota can produce, modify, and degrade metabolites within this ecosystem. We establish that cervicovaginal metabolomic output clusters by microbial community in pregnancy among Black individuals, revealing increased metabolism within the amino acid and dipeptide pathways as hallmarks of a non-optimal microbiota. Few differences were detected in metabolomic profiles when stratified by birth outcome. The study raises the possibility that metabolites could distinguish women with greater risk of sPTB among those with similar cervicovaginal microbiota, and that metabolites within the amino acid and carbohydrate pathways may play a role in this distinction.

## Introduction

Spontaneous preterm birth (sPTB) is a major cause of neonatal morbidity and mortality, and worldwide, sequelae of prematurity account for 1.1 million deaths annually. Premature cervical remodeling is thought to represent a precursory event on the pathway to sPTB. Recent evidence suggests that the biomechanical and molecular processes of premature cervical remodeling likely result from interactions between the cervicovaginal microbiota and host immune responses^1–9^. Contemporary studies of reproductive health and disease commonly utilize hierarchical clustering of cervicovaginal microbiota into community state types (CST) based on microbiota composition^10^. CST I, II, III, and V are enriched in *Lactobacillus* species, while CST IV is relatively poor in *Lactobacillus* and comprised of a wide range of strict and facultative anaerobes. Communities lacking *Lactobacillus* are considered non-optimal^11^ and have been associated with adverse reproductive health outcomes, including increased risk of urogenital disease and sexually transmitted infections^12–18^.

Recent studies examining cervicovaginal microbiota in pregnancy have established an association between a non-optimal microbiota and sPTB^9, 19–24^. We reported outcomes from a 2000-women cohort revealing that CST IV, specific bacterial taxa, and local immune factors were significantly associated with sPTB^9^. We determined that *Lactobacillus* species and antimicrobial peptide β-defensin mitigate risk associated with certain bacterial taxa. This study further demonstrated that the prevalence of a non-optimal microbiota differed by race, as 40% of non-Hispanic Black women were colonized by CST IV early in pregnancy. While these findings are compelling in the context of known racial disparities, not all women with CST IV, regardless of race, will deliver preterm. Similarly, not all cases of sPTB occur in the setting of a non-optimal microbiota. These collective observations raise questions about other undefined factors in the cervicovaginal space that might mediate or distinguish clinical outcome in the presence of the same microbiota.

Observations within the reproductive tract, as well as those in other biological niches, support the premise that microbiota affect host organ systems through regulation of microbial metabolites^25–28^. Metabolomics is the study of sub-kilodalton biochemicals, many of which have been implicated in cellular function and structure. The cervicovaginal space is metabolically active in pregnancy, and microbiota in this ecosystem can produce, modify, and degrade metabolites^29–31^. Our group previously identified a cervicovaginal metabolomic signature in pregnancy, including distinct biochemicals associated with sPTB^29, 30^. While these early studies suggest that metabolites may provide a lens into mechanisms underlying premature cervical remodeling, they did not incorporate data on microbiota composition. Published literature on the gastrointestinal system has established that the metabolome differs within the same microbial community, in part due to genetic and dietary factors^32^. As such, it is not just the microbial composition but the functional output of the collective microbiota that drives host response. Applied to the cervicovaginal space in pregnancy, it is plausible that obstetrical outcomes and risk may be governed by the effect of microbial metabolites on host function. However, no study in pregnancy to date has examined the cervicovaginal metabolome within the same ethnic group and microbial community to determine microbiome-metabolome patterns that may identify women at greatest risk for sPTB.

Given this background, we posit that the cervicovaginal metabolome may play a critical role in modulating microbiota-immune interactions, and that specific metabolites may function as biomarkers of sPTB. We therefore sought to examine cervicovaginal metabolomic output among women within specific CSTs to identify novel metabolites and related functional pathways associated with cervical remodeling and sPTB.

## Results

### Description of participants

Obstetric and clinical data are presented in Table 1. Differences in gestational age are noted between sPTB and term pregnancies (p-value < 0.001), as expected based on study design but not between women classified as having CST I or CST IV vaginal microbiomes (p = 0.52). Demographic characteristics are otherwise similar, including incidence of prior sPTB and body mass index (BMI). All study participants are self-reported non-Hispanic Black. One participant reported vaginal intercourse and a second participant reported douching and pelvic exam in the 24 h preceding swab collection. Both participants had CST I microbial communities; one had a sPTB and the other had a term birth.

**Table 1:** Demographic characteristics by Community State Type and birth outcome (n = 40).

	Term/CST I (n = 10)	Term/CST IV (n = 10)	sPTB/CST I (n = 10)	sPTB/CST IV (n = 10)	p-value^a^
**Maternal age (years)**					0.151
< 25	5 (50.0)	4 (40.0)	4 (40.0)	0 (0.0)	
25–30	3 (30.0)	5 (50.0)	4 (40.0)	5 (50.0)	
> 30	2 (20.0)	1 (10.0)	2 (20.0)	5 (50.0)	
**BMI (categorized)**					0.316
< 25	3 (30.0)	3 (30.0)	3 (30.0)	0 (0.0)	
25–30	4 (40.0)	4 (40.0)	2 (20.0)	2 (22.2)	
> 30	3 (30.0)	3 (30.0)	5 (50.0)	7 (77.8)	
**Prior sPTB (prior sPTB 16–36.6 weeks’ gestation)**					0.477
Yes	2 (20.0)	7 (70.0)	5 (50.0)	5 (50.0)	
No	8 (80.0)	3 (30.0)	5 (50.0)	5 (50.0)	

	Term, CST IMean (SD)	Term, CST IVMean (SD)	sPTB, CST IMean (SD)	sPTB, CST IVMean (SD)	p-value
Maternal age (years)	26 (4.2)	26 (5.5)	27.5 (7.3)	30.5 (4.8)	0.244^b^
Gestational age at delivery (weeks)	39 (0.9)	38.9 (0.7)	26.5 (6.0)	22.3 (3.2)	< .001^b^
BMI (kg/m^2^)	30.5 (11.7)	28.7 (10.5)	30.4 (7.2)	36.9 (8.2)	0.284^b^
Infant Birthweight (g)	3305 (332)	3169 (278)	1231 (850)	550 (271)	< .001^b^

Table presented as n (col %).

^a^Fisher’s exact p-value unless otherwise indicated.

^b^2-way ANOVA p-value.

### The cervicovaginal metabolome is associated with cervicovaginal microbiota

**Figure 1:**
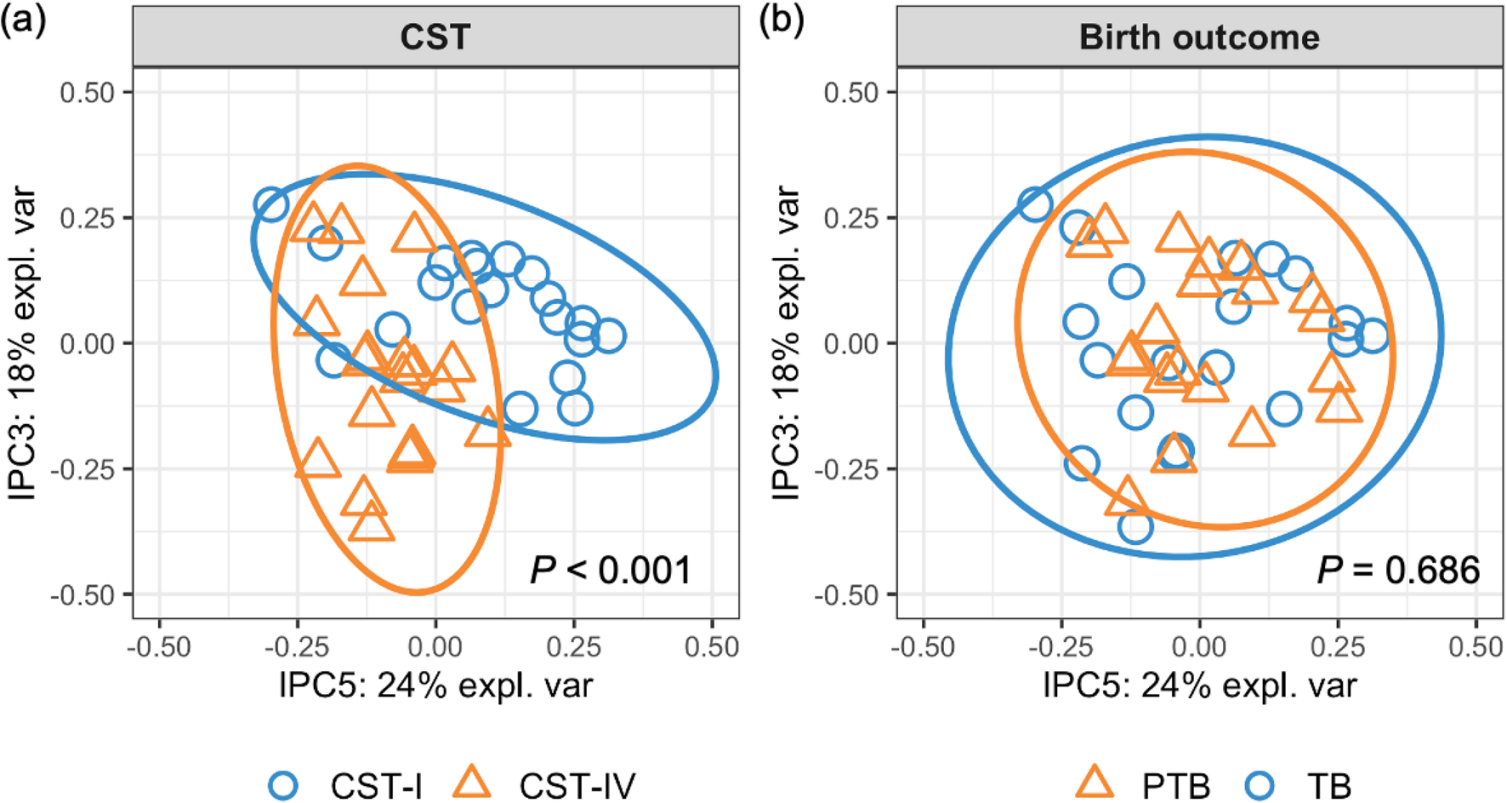
The cervicovaginal metabolome clusters by community state type (CST). Independent principal component analysis (IPCA) plots of metabolite profiles colored by CST (**a**) and birth outcome (**b**). Blue dots indicate CST I samples in (**a**) and term birth (TB) samples in (**b**); yellow triangles indicate CST IV samples in (**a**) and spontaneous preterm birth (sPTB) samples in (**b**); circles indicate the 95% confidence ellipse of samples. p values of permutational multivariate analysis of variance (PERMANOVA) are shown in each panel. The two components with the largest explained variance of metabolite composition, IPC 5 (24%) and IPC 3 (18%), are shown.

**Figure 2:**
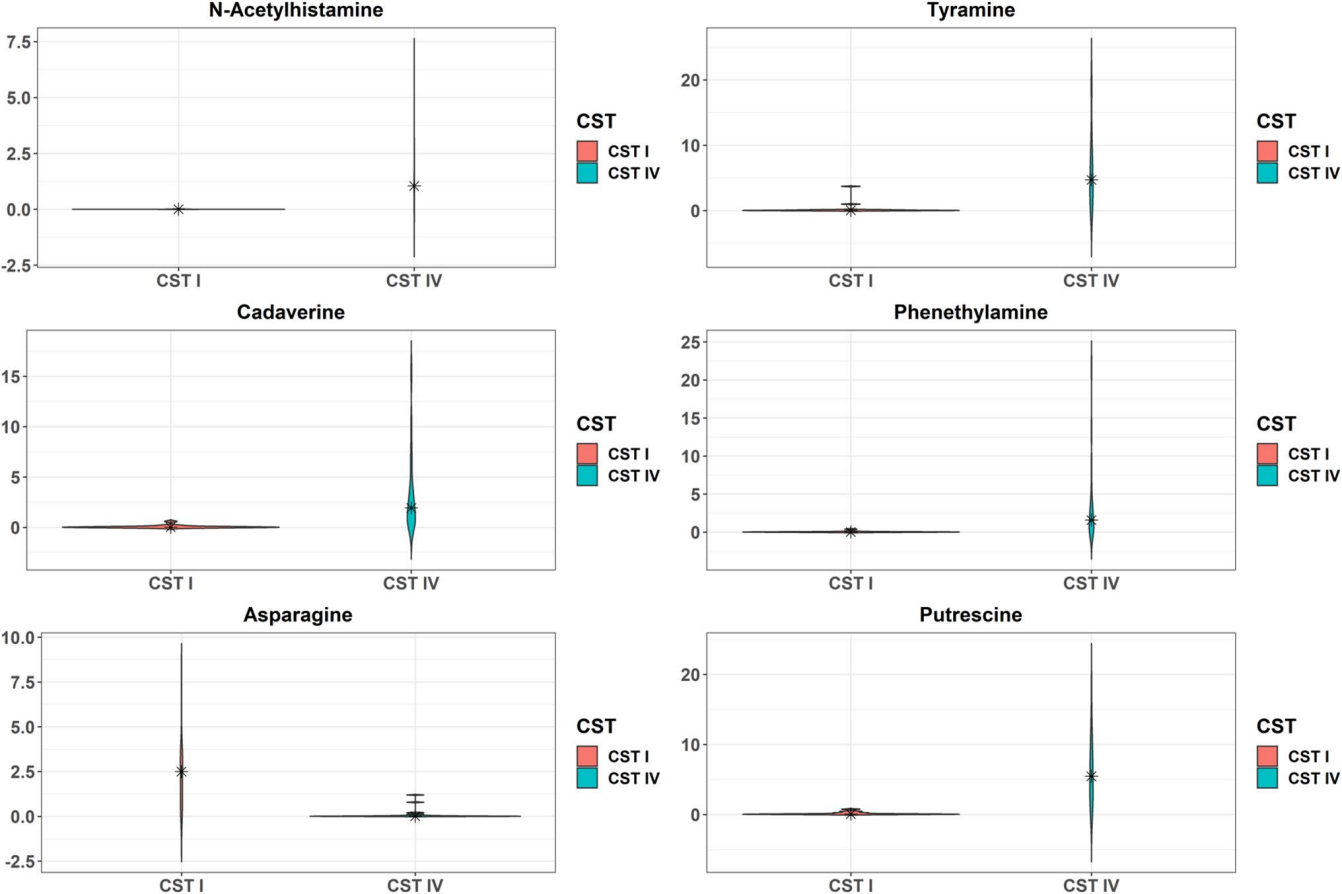
Violin plots of metabolites that differed by Community State Type (CST). Kernel probability density of various metabolites are presented. Metabolite abundances were log2 transformed and two-sided Student t-tests were performed. Resulting p-values were corrected by false discovery rates calculated using the Benjamini–Hochberg (BH) method with a threshold of ≤ 0.1. Fold change was calculated to quantify the difference in log2 change between groups for each metabolite. Metabolites with a p-value ≤ 0.05 and absolute fold changes ≥ 1.5 were considered significant. Violin plots are presented for the top six metabolites with the greatest fold change.

Analysis across groups identified 304 biochemicals present in cervicovaginal fluid. Metabolite profiles clustered primarily by CST (Fig. 1; PERMANOVA p < 0.001) rather than birth outcome (p = 0.686). A total of 133 metabolites were differentially detected among women with CST IV compared to CST I, of which 72 were increased and 61 were decreased in abundance (p ≤ 0.05, FDR ≤ 0.1, Table S1). Among the 72 upregulated metabolites, 33 (45.8%) belonged to amino acid superpathway and/or subpathways, and 11 (15.3%) belonged to carbohydrate superpathway and/or subpathways. The top six differentially detected metabolites based on absolute fold-change are presented in Fig. 2. We identified marked elevations in amino acid catabolites n-acetylhistamine (160.0-fold change, p = 2.52E−13, FDR = 2.54E−11), cadaverine (68.4-fold change, p = 7.83E−12, FDR = 3.95E−10), tyramine (56.1-fold change, p = 9.66E−10, FDR = 3.25E−8), phenethylamine (44.3-fold change, p = 2.23E−09, FDR = 6.77E−08), and putrescine (44.2-fold change, p = 4.35E−13, FDR = 3.29E−11) among participants with CST IV, while asparagine was markedly decreased (63.2-fold change, p = 5.30E−9, FDR 1.50E−7) among participants with CST IV (Fig. 2). Among downregulated metabolites, 22 (36.1%) were classified within the amino acid superpathway and/or subpathways, and 12 (19.7%) were classified within the peptide superpathway and dipeptide sub pathway.

### The cervicovaginal metabolome is associated with spontaneous preterm birth

**Figure 3:**
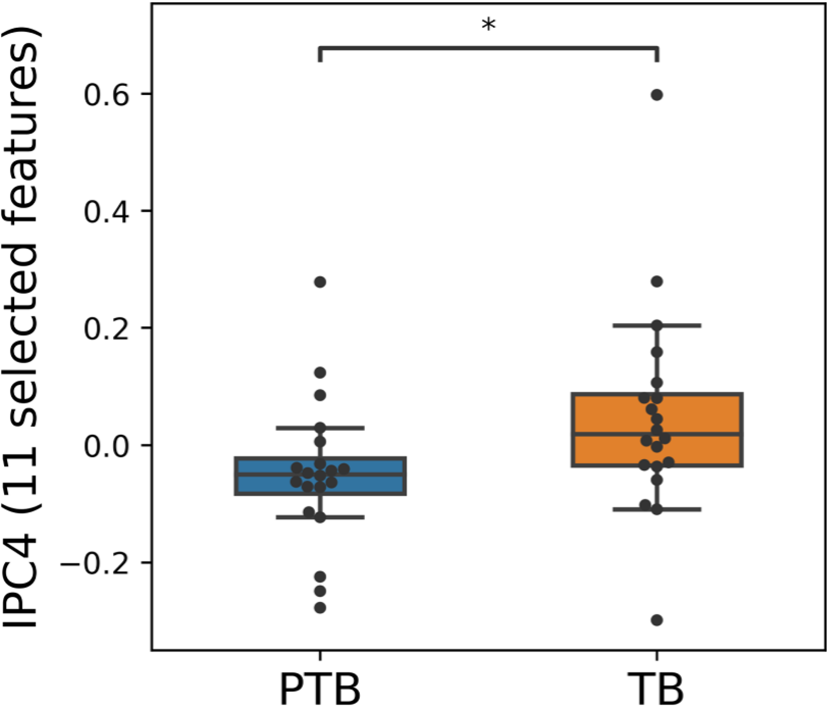
A select group of 11 metabolites in the cervicovaginal metabolome is associated with spontaneous preterm birth. Distribution of IPC4 components of women who delivered spontaneously preterm and term when 11 features are selected. Box, IQR; whiskers, 1.5*IQR; center line, median; * Mann–Whitney U p < 0.05.

We next investigated whether select groups of metabolites could distinguish between sPTB and term birth using independent principal component analysis (ICPA). We found that the 4^th^ component in IPCA (IPC4; 9% explained variance) was significantly associated with birth outcome (t-test p < 0.05) rather than with CST (t-test p = 0.410, auROC = 0.68; Table 2). To identify features (i.e. metabolites) important to IPC4, we employed sIPCA^33^ for feature selection and selected 11 features. Based on these 11 features, the IPC4 values of sPTB were significantly different from those of term birth (Mann–Whitney *U* p = 0.016; Fig. 3). Table 3 lists these 11 metabolites important to IPC4, sorted by their loadings. The top three important metabolites were identified as stearoyl-linoleoyl-glycerol, palmitoyl dihydrosphingomyelin, and nervonate. These results suggest that the combined effect or interaction of these 11 metabolites important to IPC4 might play a role in the birth outcome.

**Table 2:** Prediction of birth outcome and Community State Type (CST) using the 5 independent components (IPC1-5) and the difference of IPC1-5 by birth outcome and by CST.

Component	Birth outcome		CST	
	auROC^a^	*p*^b^	auROC^a^	*p*^b^
IPC1	0.46	0.8103	0.62	0.0599
IPC2	0.53	0.6542	0.71	0.0547
IPC3	0.52	0.7777	0.76	**0.0033**
IPC4	0.68	**0.0326**	0.55	0.4095
IPC5	0.54	0.7140	0.82	**0.0002**

^a^auROC: Area under the receiver operating characteristics.

^b^t-test *P* values for components significantly differing by birth outcome and by CST (*p* < 0.05) are bolded.

**Table 3:** List of 11 metabolites composing IPC4, associated with sPTB.

Metabolites	Loading
Stearoyl-linoleoyl-glycerol (18:0/18:2)^2^*	0.00452
Palmitoyl dihydrosphingomyelin (d18:0/16:0)*	0.00322
Nervonate (24:1n9)*	0.00268
N-stearoyl-sphinganine (d18:0/18:0)*	0.00228
1-Stearoyl-2-oleoyl-GPC (18:0/18:1)	0.00167
N-palmitoyl-sphinganine (d18:0/16:0)	0.00132
Erucate (22:1n9)	0.00081
1-Oleoyl-2-linoleoyl-GPC (18:1/18:2)*	0.00064
Glycosyl-N-palmitoyl-sphingosine (d18:1/16:0)	0.00029
Isoleucylglycine	0.00026
N-acetylisoleucine	0.00003

**Figure 4:**
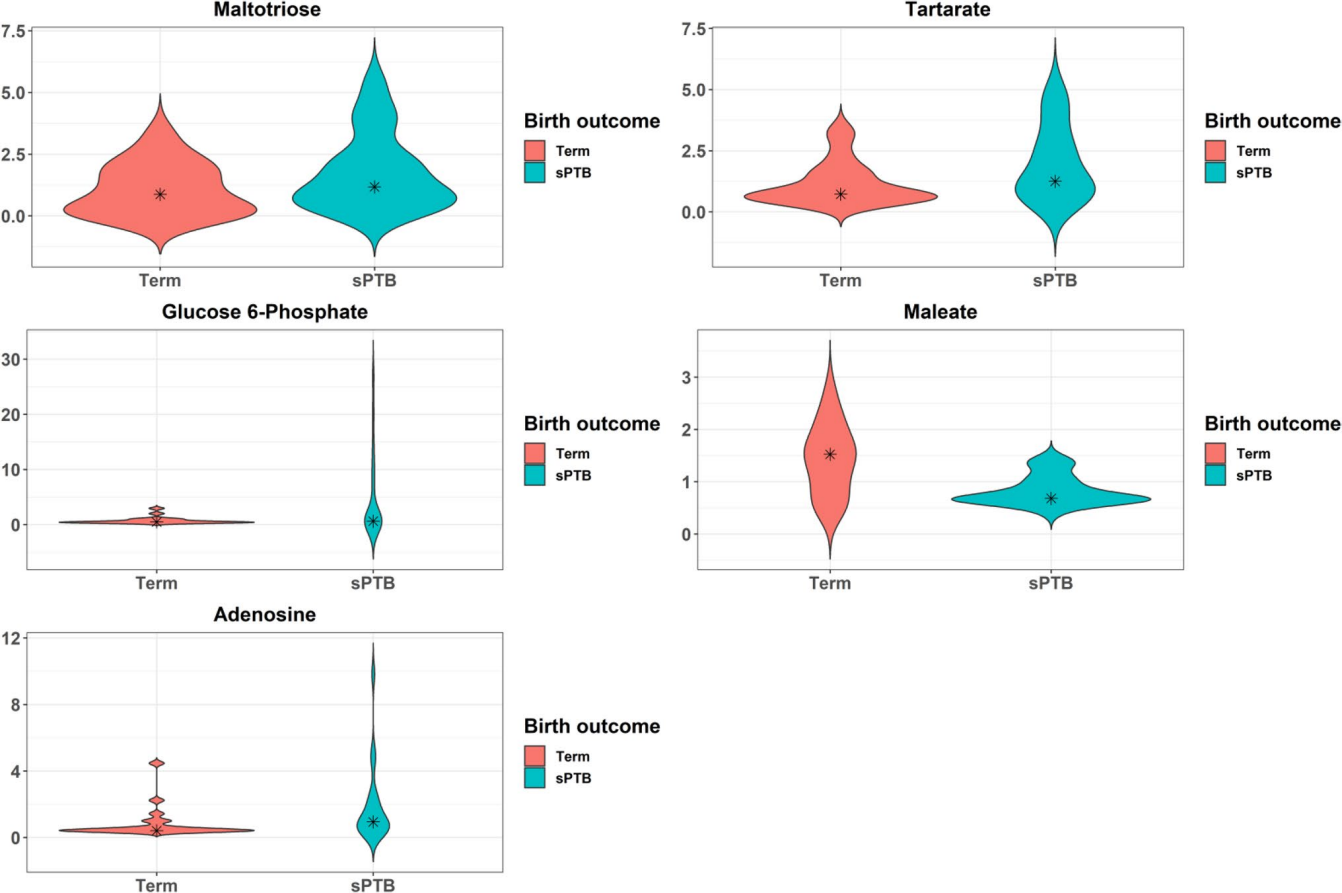
Violin plots of metabolites that differed by birth outcome. Kernel probability density of various metabolites are presented. Metabolite abundances were log2 transformed and two-sided Student t-tests were performed. Resulting p-values were corrected by false discovery rates calculated using the Benjamini–Hochberg (BH) method with a threshold of ≤ 0.1. Fold change was calculated to quantify the difference in log2 change between groups for each metabolite. Metabolites with a p-value ≤ 0.05 and absolute fold changes ≥ 1.5 were considered significant.

We next evaluated whether specific metabolites were associated with birth outcome. Five metabolites differed among women with sPTB versus term birth, among which four were increased in abundance in cases of sPTB (Fig. 4; Table S2), although none were significant post FDR correction (FDR > 0.1). Two metabolites within the carbohydrate superpathway were elevated, namely maltotriose (2.8-fold change) and glucose 6-phosphate (2.4-fold change). Maleate, a metabolite within the lipid superpathway, was decreased (1.6-fold change).

### Differences in the cervicovaginal metabolome by birth outcome within common microbial communities

**Figure 5:**
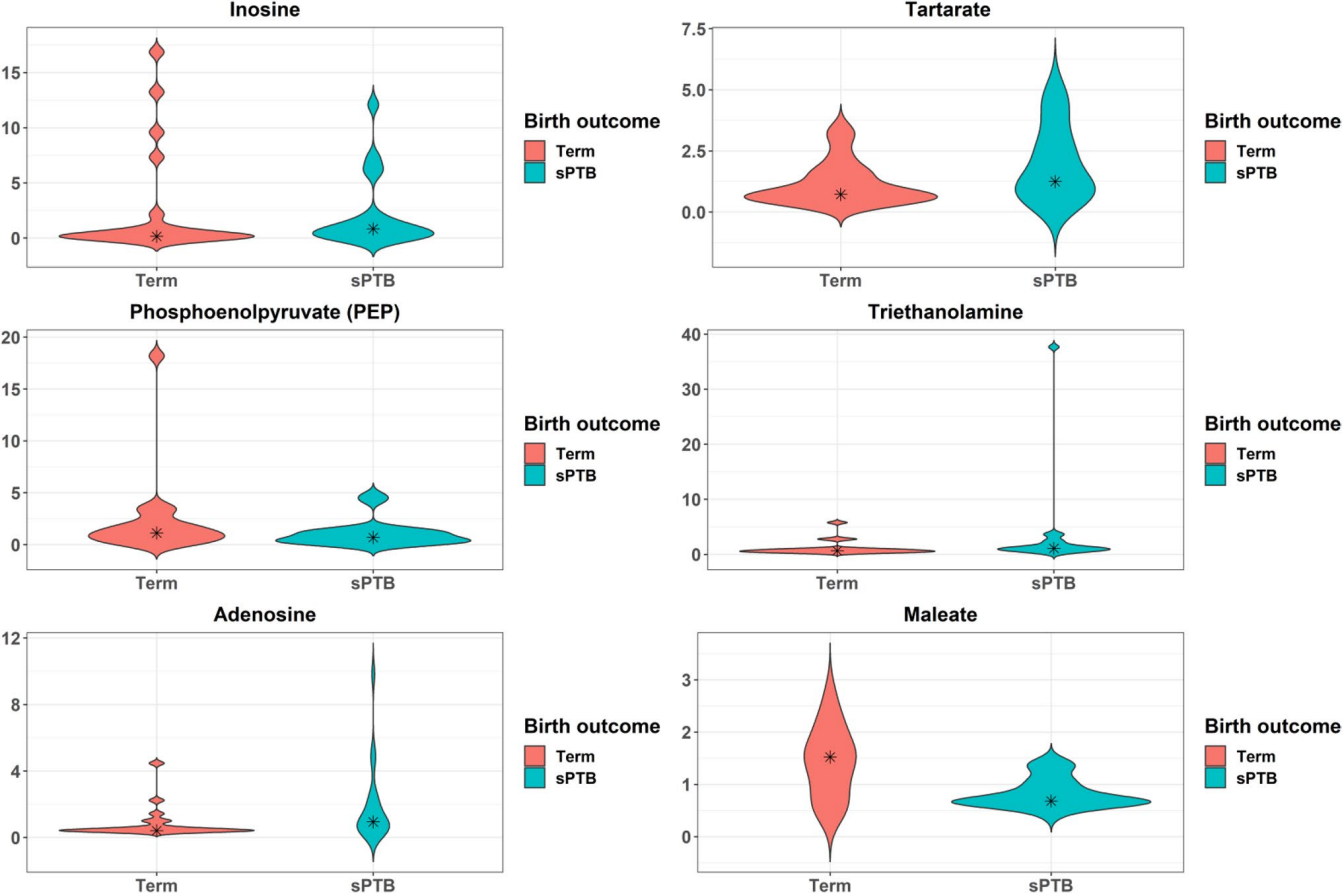
Violin plots of metabolites among CST I that differed by birth outcome. Kernel probability density of various metabolites are presented. Metabolite abundances were log2 transformed and two-sided Student t-tests were performed. Resulting p-values were corrected by false discovery rates calculated using the Benjamini–Hochberg (BH) method with a threshold of ≤ 0.1. Fold change was calculated to quantify the difference in log2 change between groups for each metabolite. Metabolites with a p-value ≤ 0.05 and absolute fold changes ≥ 1.5 were considered significant. Violin plots are presented for the top six metabolites with the greatest fold change.

We performed analyses to address the possibility that the metabolomic profile differs by birth outcome within cervicovaginal CST. We first compared metabolites abundances in CST I women who delivered spontaneously preterm or at term. We identified 10 metabolites that differed by birth outcome among women with CST I, four of which were increased in abundance in cases of sPTB (Fig. 5, Table S3). Among the six metabolites that were decreased in cases of sPTB, four metabolites belonged to the lipid superpathway. We specifically identified a decrease in lipid superpathway metabolites phosphoenolpyruvate (2.7-fold change) and maleate (1.6-fold change). After FDR correction (FDR > 0.1), however, none of these differences in relative metabolite abundances remained significant.

**Figure 6:**
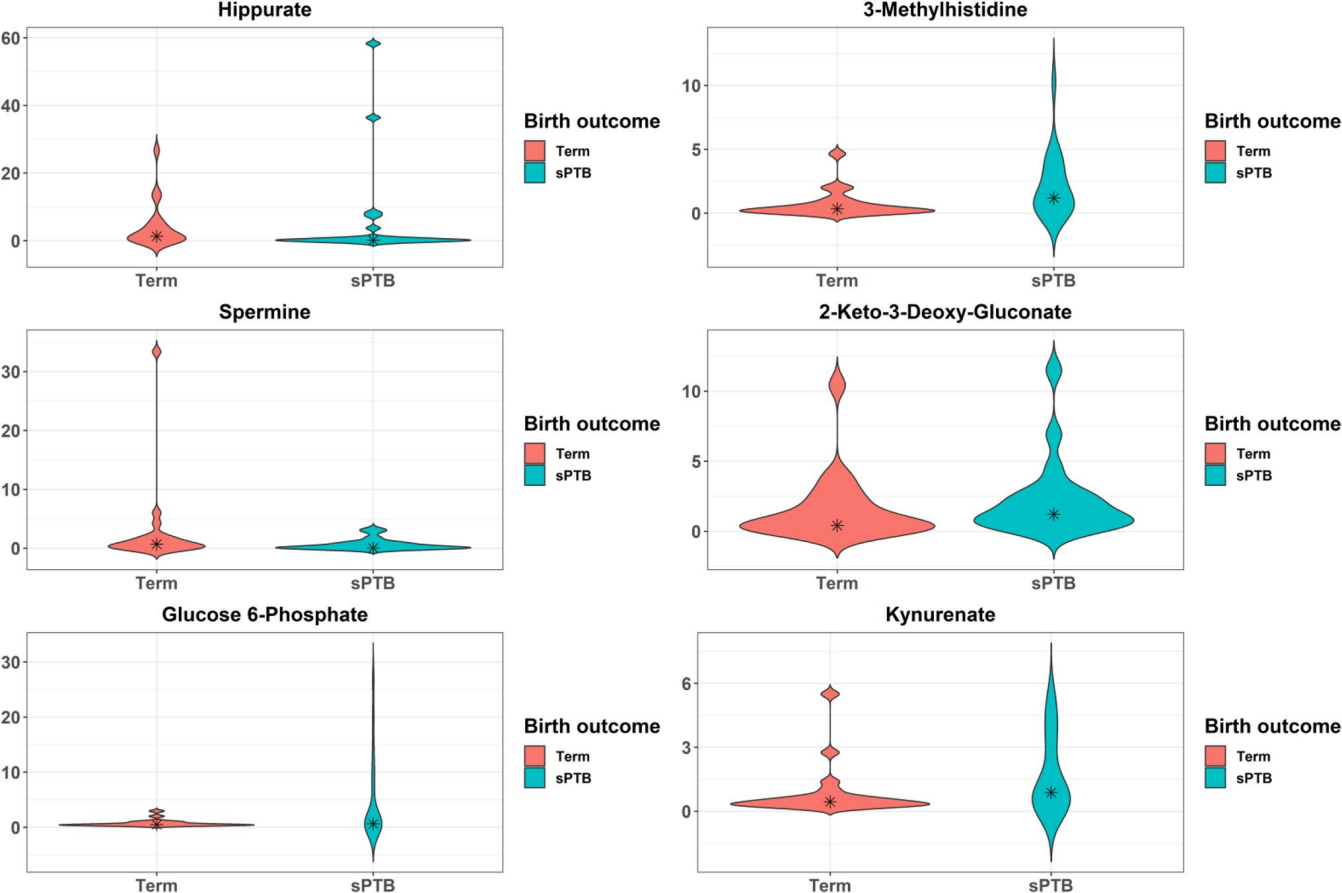
Violin plots of metabolites among CST IV that differed by birth outcome. Kernel probability density of various metabolites are presented. Metabolite abundances were log2 transformed and two-sided Student t-tests were performed. Resulting p-values were corrected by false discovery rates calculated using the Benjamini–Hochberg (BH) method with a threshold of ≤ 0.1. Fold change was calculated to quantify the difference in log2 change between groups for each metabolite. Metabolites with a p-value ≤ 0.05 and absolute fold changes ≥ 1.5 were considered significant. Violin plots are presented for the top six metabolites with the greatest fold change.

To determine whether within non-optimal cervicovaginal microbiota (CST IV), a known risk factor for sPTB, metabolites could identify women at greatest risk of early delivery, we compared metabolite abundances in CST IV women who delivered spontaneously preterm or at term. There were 13 differentially abundant metabolites, the top six of which based on absolute fold change are presented in Fig. 6 (see also Table S4). Among the 13 metabolites, 10 were increased in sPTB compared to term birth. Those with the greatest fold change included glucose-6-phosphate (6.7-fold change), 3-methylhistidine (6.4-fold change), 2-keto-3-deoxygluconate (3.2-fold change), and kynurenate (3.0-fold change). Among these 10 metabolites, four belonged to the carbohydrate superpathway and four belonged to the amino acid superpathway. The three metabolites that were decreased in abundance in cases of sPTB compared to term birth included hippurate (9.1-fold change), spermine (6.9-fold change), and maleate (1.6-fold change) (p < 0.05 for all). After FDR correction (FDR > 0.1), however, none of these differences in relative metabolite abundances remained significant.

## Discussion

Our analyses establish that cervicovaginal metabolomic output clusters by microbial community in pregnancy among individuals self-identifying as Black, and reveal increased metabolism within the amino acid and dipeptide superpathways as hallmarks of CST IV compared to CST I. To our knowledge, this study is examining for the first time the cervicovaginal metabolome in Black individuals and stratified by cervicovaginal microbiota. Relatively few differences were detected in metabolomic profiles when stratified by birth outcome. After correction for multiple comparisons, we did not detect significant differences in metabolomic profiles by birth outcome within the same CST though the small sample size limit our ability to draw conclusions from these subanalyses. Nonetheless, our study raises the possibility that metabolites, in particular those within the amino acid and carbohydrate superpathways could potentially distinguish women with greater risk of sPTB among those with similar cervicovaginal CSTs.

Findings from this study highlight the importance of the amino acid superpathway in distinguishing metabolomic output between an optimal (CST I) and non-optimal (CST IV) microbiota. Our observations support the concept that anaerobic organisms preferentially utilize amino acids as carbon and nitrogen energy sources, in contrast to *Lactobacillus* species, thereby leading to increased abundance of amino acid catabolites. Alternatively, anaerobes may produce greater amounts of amino acids, which they subsequently convert to biogenic amines (such as cadaverine and tyramine as presented in Fig. 2) or other metabolites. While the microbiota of bacterial vaginosis (BV) is heterogeneous, specific bacterial species common to this infection are present in CST IV, and our findings are congruent with data characterizing the cervicovaginal metabolome among non-pregnant women with BV^27, 34^. These studies have identified similar increased abundance of amino acid catabolites and polyamines in BV, some of which may affect signaling cascades and trigger host inflammation or disrupt epithelial barrier integrity.

It is worth noting that metabolites in the amino acid superpathway were both significantly upregulated and downregulated when comparing optimal to non-optimal cervicovaginal microbiota, and this observation may reflect physiological shifts to maintain homeostasis in response to distinct microenvironments. Metabolites that are increased may counteract those that are decreased in abundance, thereby providing metabolomic equipoise. An alternative and perhaps more biologically plausible explanation, especially in light of our understanding that select cervicovaginal microbiota confer differential risk of adverse pregnancy outcomes, is that these observed alterations in the relative abundance of metabolites in the amino acid subpathway generate relatively unopposed bioactive metabolites. These metabolites may induce host inflammation or barrier disruption in the absence of protective metabolites or factors. For example, we identified a 3.8-fold decrease in tryptophan among participants with CST IV compared to CST I. Conversely, breakdown products of tryptophan, including tryptamine and kynurenate, were among those significantly increased metabolites when comparing CST IV to CST I. Tryptamine is a biogenic amine that has been associated with CST IV cervicovaginal microbiota and negatively correlates with *Lactobacillus* abundance^35^. Tryptamine also appears to mediate key physiologic functions of the gastrointestinal tract including epithelial secretion, which is important for host functions such as regulating immune response to pathogenic organisms and maintaining luminal fluid concentrations^36, 37^. Among women with a non-optimal microbiota who go on to sPTB, only kynurenate (and not tryptamine) is significantly increased in abundance prior to FDR correction, raising the possibility that a relative lower abundance of tryptamine in a CST IV community may compromise host immune surveillance or function of the cervicovaginal epithelium. It is plausible that tryptamine confers some degree of host protection in the setting of high-risk cervicovaginal microbiota, and that this defense is lacking in women with CST IV who delivery spontaneously preterm. Notably, perturbations in the kynurenine pathway of tryptophan metabolism have been linked to inflammatory conditions, cardiovascular disorders, and neurodegenerative disease^38^. Our observations raise the possibility that tryptophan metabolites, including tryptamine and kynurenate, may regulate host immune response and potentially serve as mediators of cervical remodeling. As these metabolites have yet to be explored in the cervicovaginal space, this hypothesis warrants mechanistic investigation in future studies.

With respect to stabilization of epithelial barriers, another interesting finding is the decrease in the polyamine spermine among women with a non-optimal microbiota and sPTB prior to FDR correction. Notably, our group has established that microbial supernatants from select high-risk vaginal bacteria associated with a non-optimal microbiota can disrupt the cervical epithelial barrier in vitro, and that this process involves cleavage of cell adhesion protein E-cadherin^31, 39^. Spermine is commonly associated with *Lactobacillus*-dominated cervicovaginal microbial communities and has been shown to increase production of lactic acid by *Lactobacillus*^40^. As lactic acid can function in an anti-inflammatory capacity in this space^41–44^, it is possible that spermine plays a major role at the cervicovaginal epithelium. In other systems, polyamines have been shown to regulate epithelial barrier function, specifically through modulation of cell adhesion proteins, including E-cadherin and occludin, as well select signaling pathways regulating the expression of these proteins^45–47^. In intestinal epithelial cells, spermine has been shown to prevent disassembly of focal adhesions required for cell migration by binding to the SH2 domain of Src^48^. These observations collectively raise the possibility that decreased abundance of spermine in the cervicovaginal space impedes epithelial barrier stabilization and/or promotes barrier permeability, thus contributing to cervical remodeling.

Our findings raise questions as to the role of increased glycolytic intermediates, including glucose, glucose 6-phosphate, pyruvate, and 2-phosphoglycerate in women with a non-optimal compared to optimal microbiota. In the vagina, *Lactobacillus* grows under anaerobic conditions and can convert pyruvate to lactic acid. Under aerobic conditions, however, *Lactobacillus* utilizes lactic acid to generate pyruvate, thereby increasing vaginal pH. This physiology is consistent with our observation that pyruvate abundance is significantly higher among women with CST IV. Though not statistically significant in our analyses post FDR correction, select glycolytic intermediates, including glucose 6-phosphate and pyruvate, may be further increased among women with CST IV who delivered spontaneously preterm, suggesting that cervicovaginal carbohydrate metabolism is potentially amplified in this subset of women. The role of glycolysis and microbiota has been explored in other mucosal surfaces, including the gastrointestinal tract, where increased glucose metabolism has been implicated in the breakdown of intestinal epithelial cell barriers, thereby leading to transcriptional reprogramming, inflammation, and enteric infection^49^. Our laboratory has demonstrated that specific bacterial species present in CST IV compromise the integrity of cervical epithelial cell barriers, alter immune response, and induce epigenetic changes^31^. Future studies investigating the functional and immune effects of select glycolytic intermediates on the cervicovaginal epithelium are warranted.

Another notable finding includes the predominance of lipid metabolites comprising the group of 11 metabolites associated with sPTB in the ICPA. Nine of the 11 metabolites belonged to the lipid superpathway, and all were decreased in abundance in cases of sPTB compared to term birth. Several of these metabolites are components of linolenic and arachidonic acid metabolism pathways, raising the possibility that these eicosanoid precursors are being consumed in the production of prostaglandins, which play a known role in cervical ripening and inflammation. Among the decreased metabolites are also sphingolipids, a class of lipid metabolites that stabilize plasma membranes and protect cells against harmful environmental exposures, as well as function in cell signaling and immune regulation. It is biologically plausible that this relative decrease in abundance of sphingolipids renders the cervicovaginal epithelium more vulnerable to environmental factors while potentially contributing to a maladaptive host immune response.

Our findings expand upon the published literature from our group characterizing cervicovaginal metabolomic signatures in pregnancy associated with sPTB^29, 30^. We demonstrated previously that low-risk *symptomatic* women (i.e. individuals with symptoms of preterm labor) between 22 to 34 weeks of gestation who go on to sPTB have distinct cervicovaginal biochemical output compared to controls with threatened preterm labor who go on to term deliveries^30^. Though none of the individual metabolites were overlapping between this current study and those identified in that cohort, common differentially detected superpathways were identified among women who delivered spontaneously preterm, including superpathways associated with amino acid and carbohydrate metabolism. When our current findings are compared to patterns of cervicovaginal metabolomic output identified in a cohort of *asymptomatic* women between 20 to 28 weeks of gestation at high risk of early delivery^29^, metabolic changes in the carbohydrate superpathway were detected, including increased abundance of glucose-6-phosphate, in women who delivered preterm. That study also identified differential detection of metabolites in the amino acid superpathway in cases of sPTB, consistent with our current study.

Metabolomic variance between symptomatic and asymptomatic women may be explained, at least in part, by differences in host immune response, including local and systemic inflammation. Differences between this current study and those two previously published cohorts from our laboratory may result from differences in the timing of cervicovaginal fluid sampling, as well as racial differences. The observation that increased glucose and amino acid metabolism is associated with sPTB across all three studies, however, provides compelling biologic plausibility that glycolytic intermediates and amino acid catabolites may play a key role in cervical remodeling and sPTB.

Our data suggest a critical role for the cervicovaginal metabolome in modulating microbiota-immune interactions. Identifying bioactive metabolites and defining their mechanisms of action will likely facilitate recognition of novel biomarkers and pathways through which targeted interventions can successfully block premature cervical remodeling. Cervicovaginal metabolomic profiling in pregnancy may offer potential as a tool for sPTB risk stratification, thereby enhancing existing clinical strategies such as cervical length measurement. When considered in the context of a non-optimal microbiota, metabolites may be useful to identify women at greatest risk clinically. While certain high-risk bacterial species in combination with select metabolites may induce similar biologic effecs, and therefore represent the same hit to the system, it is possible that the presence of both is additive or even synergistic. Our observations also carry significant translational implications; metabolites offer potential as therapeutics in maintaining or restoring cervicovaginal homeostasis, thereby blocking the cascade of events that lead to aberrant cervical remodeling and sPTB. Spermine may represent one such example given our observations and its known role in epithelial barrier stabilization in other microenvironments.

Strengths of this study include our ability to capture a cohort of non-Hispanic Black women with well-characterized cervicovaginal microbiota early in the second trimester. Focus on a single racial group and stratification by CST provides insight into metabolomic changes that may confer increased risk of early delivery whether the vaginal microbiota is considered optimal or non-optimal. Many published studies examining risk factors associated with sPTB have been limited by poorly phenotyped birth outcomes, resulting in clinical and biologic heterogeneity. Our pregnancy cohort is comprised of women with clear preterm birth phenotyping. Limitations of this study include the sample size of 40 women with cervicovaginal metabolomic analysis at a single time point in pregnancy; these data underscore the need for larger prospective studies characterizing metabolomic output across gestation associated with both optimal and non-optimal cervicovaginal microbial communities and birth outcome. The study is also limited by potential confounding of vaginal exposures predecing swab collection, which could impact metabolite quantification. This cohort, however, has a low prevalence of these exposures with only two participants reporting vaginal intercourse, douching, or pelvic exam within the 24 h preceding swab collection.

The cellular origin of differentially detected biochemicals remains a major unanswered question; it is unclear whether metabolites of interest are derived from host cells, specific microbial species, or both. Based on our understanding of the microbiome-metabolome interface in other biological systems in the context of health and disease^50^, it is likely that both host- and microbiota-derived metabolites play a biologically active role in the cervicovaginal space. It is reasonable to conclude that biochemicals belonging to the xenobiotic superpathway arise from microbiota or dietary intake rather than eukaryotic cells. While metabolites of bacterial origin may induce pathologic changes in mucosal immunity and epithelial barrier integrity, these small molecules may also be inhibitory to other bacterial species and alter microbial homeostasis within the cervicovaginal space^40^.

Future mechanistic studies involving in vitro models may unveil pathways through which select metabolites contribute to premature cervical remodeling and sPTB. The ability of such biochemicals to inhibit or promote growth of commensal microbiota is of particular interest. Conversely, the potential for healthy microbial species to modify metabolomic signatures associated with adverse outcomes, as well as the role of host immune response in mediating these processes, warrant further investigation.

## Methods

### Study setting

A nested case–control study was performed from a prospective cohort study entitled *Motherhood and Microbiome (M&M)* in which 2,000 women enrolled from December 2013 through February 2017 as previously described^9^. The Institutional Review Board at the University of Pennsylvania approved this study. All methods were performed in accordance with the relevant guidelines and regulations. Informed consent was obtained from all subjects. Exclusion criteria included a major fetal anomaly, HIV seropositive status, history of organ transplant, chronic steroid use, enrollment into the study during a previous pregnancy, or multiple gestations. Particiants were asked whether they had vaginal intercourse, douched, or had a pelvic exam in the 24 h preceding cervical swab collection. Cervical swabs were collected at 16 to 20 weeks of gestation and cervicovaginal microbiota were analyzed by 16S rRNA gene sequencing^9^. Microbial communities were classified into CST as previously reported using hierarchical clustering with Jensen-Shannon divergence and Ward linkage^10^. CST I is predominated with *L. crispatus*, CST II with *L. gasseri*, CST III with *L. iners* and CST V with *L. jensenii*. CST IV is defined by a paucity of *Lactobacillus* species and a diverse set of strict and facultative anaerobes. Cases of preterm birth (PTB) were individually adjudicated by a maternal–fetal medicine physician (MAE) to distinguish spontaneous from medically indicated. PTB was considered spontaneous when a woman presented with either cervical dilation and/or premature rupture of membranes and delivered prior to 37 weeks of gestation.

Inclusion criteria for this nested case–control study was self-reported non-Hispanic Black race and 16S rRNA gene data from cervicovaginal samples obtained at 16–20 weeks of gestation. Twenty sPTB and term birth control pairs were frequency-matched by CST. Four groups of 10 women were analyzed: CST I/sPTB; CST I/term birth; CST IV/sPTB; and CST IV/term birth. Only women who delivered between 38 to 40 weeks of gestation were included in the term birth group to avoid misclassification.

### Biospecimen collection

Cervicovaginal fluid was collected following insertion of a sterile speculum using a sterile cotton-tipped swab. Samples were collected in 0.5 mL phosphate-buffered saline, immediately placed in liquid nitrogen, and stored at − 80 °C for future use.

### Metabolomics analysis

Metabolomics analysis was performed by Metabolon®, Inc. (Research Triangle Park, NC) as previously described^51^, and were analyzed by both Ultra-Performance Liquid Chromatography/tandem mass spectrometry (UPLC-MS/MS;Waters ACQUITY UPLC and Thermo-Finnigan LTQ mass spectrometer) and Gas Chromatography/Mass Spectrometry (GC/MS; Thermo-Finnigan Trace DSQ fast-scanning single-quadrupole mass mectrometer).

Raw data was extracted, peak-identified and quality control processed using Metabolon's hardware and software^52^. Compounds were identified by comparison to library entries of purified standards or recurrent unknown entities and assigned to key metabolic pathway groups, including amino acids, carbohydrates, cofactors and vitamins, energy, lipids, nucleotides, peptides, and xenobiotic metabolism.

### Statistical analyses

#### Demographics

Bivariate comparisons of maternal characteristics were performed between cases of sPTB and term birth controls using Chi-square (χ2) or Fisher's exact test, where appropriate. Continuous variables were compared by the Student t-test or the Mann–Whitney U test, depending on the distribution of the data.

#### Independent principal component analysis (IPCA)

Metabolite measurements were volume normalized, followed by robust standardization of the log_10_ transformed values (subtracting the median and dividing by the standard deviation calculated while clipping the top and bottom 5% of outliers). Metabolite measurements of zero were imputed using the minimum transformed value across all samples. Similar transformations have previously yielded excellent agreement with measurements performed by an independent certified medical laboratory^53^. IPCA was run using the ipca function of the R mixOmics package^33^. Permutational multivariate analysis of variance (PERMANOVA)^54^ was employed to test whether the centroids of samples as grouped by CST or birth outcome are equivalent based on Bray–Curtis distance, using the adonis function of the R vegan package. Area under the receiver operating characteristics (auROC) for the prediction of CST and birth outcome using the five IPCs identified in IPCA was measured. Independent t-tests were employed to test the difference of IPCs by birth outcome and by CST. Sparse IPCA (sIPCA)^33^ was further conducted to select features for IPCs using the sipca function of the R mixOmics package. We assessed the relationship between the number of features selected and the difference of IPC4 between sPTB and term birth measured by the p values of Mann–Whitney *U* tests. The optimal features selected would be a set of minimum features which yield the most significant difference in IPC4 components between sPTB and term birth. The optimal number of selected features was determined as 11 (Fig. S1).

#### Differences between CSTs

The primary metabolomics analysis was conducted on metabolite abundance data that was volume-normalized to protein content. All data were log_2_ transformed for comparison. Median metabolite abundance was compared by CST (I versus IV) and by birth outcome (sPTB versus term). Sensitivity analyses were conducted comparing median metabolite abundance by birth outcome among CSTs. Two-sided Student t-tests were used for comparison, and resulting p-values were corrected by false discovery rates calculated using the Benjamini–Hochberg (BH) method with a threshold of ≤ 0.1. Fold change was calculated to quantify the difference in log_2_ change between groups for each metabolite. Only metabolites with a p-value ≤ 0.05 and absolute fold changes ≥ 1.5 were considered significant. Clinical data were analyzed using STATA (v14.2; StataCorp, Inc., College Station, TX). An estimate of the false discovery rate (q-value) was calculated to account for multiple comparisons^55^.

## Supplementary Information

Supplementary Information.

## Data Availability

The datasets generated during and/or analysed during the current study are available from the corresponding author on reasonable request.
