# Supplementary material for: A non-optimal cervicovaginal microbiota in pregnancy is associated with a distinct metabolomic signature among non-Hispanic Black individuals
Source: Sci Rep. 2021 Nov 23;11:22794. doi: 10.1038/s41598-021-02304-0 (PMC8611022; doi:10.1038/s41598-021-02304-0)
Supplement: Supplementary file 1 — Supplementary Information. [file 41598_2021_2304_MOESM1_ESM.docx]

SUPPLEMENTAL TABLES

| **Supplemental Table 1. Metabolic profile by Community State Type (n=40)** | | | | | | | |
| --- | --- | --- | --- | --- | --- | --- | --- |
| **Metabolite** | **Super Pathway** | **Sub Pathway** | **CST I Median (IQR) (n=20)** | **CST IV Median (IQR) (n=20)** | **Fold change** | **p-value** | **q-value** |
| n-acetylhistamine | Amino Acid | Histidine Metabolism | 0 (0-0) | 1.04 (0.43-2.57) | 160.01 | 2.52E-13 | 2.54E-11 |
| cadaverine | Amino Acid | Lysine Metabolism | 0.01 (0.01-0.01) | 1.93 (0.77-3.93) | 68.36 | 7.83E-12 | 3.95E-10 |
| tyramine | Amino Acid | Tyrosine Metabolism | 0.05 (0.01-0.13) | 4.70 (2.13-8.82) | 56.06 | 9.66E-10 | 3.25E-08 |
| phenethylamine | Amino Acid | Phenylalanine Metabolism | 0.02 (0.02-0.02) | 1.58 (0.21-3.58) | 44.32 | 2.23E-09 | 6.77E-08 |
| putrescine | Amino Acid | Polyamine Metabolism | 0.08 (0.03-0.19) | 5.46 (2.17-8.9) | 44.23 | 4.35E-13 | 3.29E-11 |
| 1-methylhistamine | Amino Acid | Histidine Metabolism | 0.01 (0.01-0.04) | 1.43 (0.75-2.12) | 41.3 | 2.75E-12 | 1.67E-10 |
| alpha-hydroxyisovalerate | Amino Acid | Leucine, Isoleucine and Valine Metabolism | 0.29 (0.25-0.48) | 15.72 (9.12-27.77) | 37.25 | 3.75E-15 | 1.13E-12 |
| histamine | Amino Acid | Histidine Metabolism | 0.01 (0.01-0.23) | 2.49 (0.85-3.9) | 35.65 | 1.54E-08 | 3.90E-07 |
| deoxycarnitine | Lipid | Carnitine Metabolism | 0.03 (0.03-0.07) | 2.61 (1-3.65) | 33.94 | 2.16E-13 | 2.54E-11 |
| n-acetyl-cadaverine | Amino Acid | Lysine Metabolism | 0.02 (0.02-0.06) | 1.33 (0.72-4.08) | 33.61 | 5.99E-10 | 2.27E-08 |
| 2-hydroxy-3-methylvalerate | Amino Acid | Leucine, Isoleucine and Valine Metabolism | 0.03 (0.03-0.08) | 1.59 (0.97-3.39) | 27.74 | 1.21E-11 | 5.22E-10 |
| imidazole propionate | Amino Acid | Histidine Metabolism | 0.02 (0.02-0.14) | 1.89 (0.25-9.96) | 26.61 | 6.40E-07 | 7.40E-06 |
| 5-aminovalerate | Amino Acid | Lysine Metabolism | 0.03 (0.03-0.03) | 1.88 (0.18-4.05) | 22.52 | 1.90E-07 | 3.20E-06 |
| n-acetylputrescine | Amino Acid | Polyamine Metabolism | 0.18 (0.06-0.39) | 4.47 (1.54-7.62) | 19.73 | 3.68E-08 | 7.40E-07 |
| xanthine | Nucleotide | Purine Metabolism, (Hypo)Xanthine/Inosine containing | 0.2 (0.09-0.54) | 4.71 (1.12-27.98) | 18.35 | 4.50E-06 | 4.40E-05 |
| nicotinate | Cofactors and Vitamins | Nicotinate and Nicotinamide Metabolism | 0.08 (0.04-0.25) | 3.56 (0.76-6.63) | 16.21 | 1.70E-06 | 1.80E-05 |
| pipecolate | Amino Acid | Lysine Metabolism | 0.18 (0.1-0.42) | 4.78 (1.55-6.04) | 13.52 | 7.42E-08 | 1.40E-06 |
| n-acetylneuraminate | Carbohydrate | Aminosugar Metabolism | 0.08 (0.04-0.47) | 2.01 (1-3.23) | 12.13 | 4.80E-07 | 6.10E-06 |
| uracil | Nucleotide | Pyrimidine Metabolism, Uracil containing | 0.18 (0.18-0.37) | 1.45 (0.61-11.54) | 9.22 | 1.60E-05 | 1.10E-04 |
| 2,3-dihydroxy-2-methylbutyrate | Amino Acid | Leucine, Isoleucine and Valine Metabolism | 0.09 (0.09-0.09) | 0.96 (0.13-2.78) | 8.11 | 4.60E-05 | 3.20E-04 |
| tryptamine | Amino Acid | Tryptophan Metabolism | 0.03 (0.03-0.03) | 0.35 (0.04-1.73) | 7.94 | 1.40E-04 | 8.10E-04 |
| thymine | Nucleotide | Pyrimidine Metabolism, Thymine containing | 0.41 (0.25-0.63) | 3.6 (1.13-12.02) | 7.09 | 8.40E-06 | 7.30E-05 |
| 2-hydroxybutyrate/2-hydroxyisobutyrate | Amino Acid | Glutathione Metabolism | 0.34 (0.14-0.5) | 2.14 (1.2-3.36) | 5.66 | 1.75E-08 | 4.10E-07 |
| nicotinamide | Cofactors and Vitamins | Nicotinate and Nicotinamide Metabolism | 0.59 (0.23-0.88) | 3.4 (1.4-5.68) | 5.03 | 8.90E-05 | 5.60E-04 |
| trigonelline (n'-methylnicotinate) | Cofactors and Vitamins | Nicotinate and Nicotinamide Metabolism | 0.3 (0.18-1) | 2.51 (1.28-5.83) | 4.88 | 1.10E-03 | 4.90E-03 |
| alpha-hydroxyisocaproate | Amino Acid | Leucine, Isoleucine and Valine Metabolism | 0.3 (0.19-0.54) | 2.1 (1.21-2.98) | 4.8 | 5.70E-06 | 5.20E-05 |
| glucose | Carbohydrate | Glycolysis, Gluconeogenesis, and Pyruvate Metabolism | 0.56 (0.29-0.88) | 3.54 (1.3-4.88) | 4.74 | 9.40E-05 | 5.80E-04 |
| 2-hydroxyglutarate | Lipid | Fatty Acid, Dicarboxylate | 0.65 (0.28-1.16) | 2.14 (0.98-9.36) | 4.74 | 4.70E-05 | 3.20E-04 |
| n6-acetyllysine | Amino Acid | Lysine Metabolism | 0.08 (0.08-0.08) | 0.84 (0.13-1.46) | 4.39 | 1.20E-04 | 7.60E-04 |
| n6,n6-dimethyllysine | Amino Acid | Lysine Metabolism | 0.11 (0.11-0.17) | 1.01 (0.17-1.91) | 4.32 | 1.80E-04 | 9.60E-04 |
| 13-hode + 9-hode | Lipid | Fatty Acid, Monohydroxy | 0.24 (0.24-0.24) | 1.21 (0.69-2.02) | 4.03 | 3.90E-07 | 5.20E-06 |
| 5-methylthioadenosine (mta) | Amino Acid | Polyamine Metabolism | 0.19 (0.19-0.55) | 1.25 (0.82-2.23) | 3.83 | 1.30E-05 | 1.00E-04 |
| succinate | Energy | TCA Cycle | 0.63 (0.43-0.99) | 2.51 (1.42-6.12) | 3.73 | 1.80E-04 | 9.70E-04 |
| 1-methyladenine | Nucleotide | Purine Metabolism, Adenine containing | 0.2 (0.17-0.48) | 1.7 (0.47-2.82) | 3.48 | 1.50E-04 | 8.70E-04 |
| n6,n6,n6-trimethyllysine | Amino Acid | Lysine Metabolism | 0.32 (0.06-0.92) | 1.4 (0.45-2.63) | 3.24 | 9.30E-03 | 0.030 |
| n-acetylalanine | Amino Acid | Alanine and Aspartate Metabolism | 0.66 (0.31-1.28) | 2.41 (0.9-4.24) | 3.19 | 1.80E-03 | 7.00E-03 |
| 2-isopropylmalate | Xenobiotics | Food Component/Plant | 0.2 (0.2-0.71) | 1 (0.42-3.01) | 3.15 | 3.60E-03 | 0.010 |
| fumarate | Energy | TCA Cycle | 0.28 (0.28-0.55) | 1.11 (0.85-2.33) | 3.15 | 7.50E-06 | 6.70E-05 |
| n-acetylleucine | Amino Acid | Leucine, Isoleucine and Valine Metabolism | 0.32 (0.19-0.55) | 1.1 (0.53-2.28) | 3.05 | 8.20E-04 | 3.80E-03 |
| 7-methylguanine | Nucleotide | Purine Metabolism, Guanine containing | 0.13 (0.13-0.36) | 0.73 (0.34-2.75) | 3.03 | 4.00E-03 | 0.010 |
| fructose | Carbohydrate | Fructose, Mannose and Galactose Metabolism | 0.21 (0.21-0.38) | 1.05 (0.4-1.9) | 2.98 | 3.00E-04 | 1.50E-03 |
| n-acetyltaurine | Amino Acid | Methionine, Cysteine, SAM and Taurine Metabolism | 0.25 (0.16-0.42) | 1.23 (0.38-1.78) | 2.95 | 6.40E-04 | 3.00E-03 |
| 2-piperidinone | Xenobiotics | Food Component/Plant | 0.3 (0.3-0.3) | 0.58 (0.3-2.49) | 2.79 | 5.60E-03 | 0.020 |
| n-acetylglutamate | Amino Acid | Glutamate Metabolism | 0.39 (0.13-0.88) | 1.75 (0.53-3.29) | 2.75 | 0.010 | 0.030 |
| phosphoethanolamine | Lipid | Phospholipid Metabolism | 0.19 (0.13-0.69) | 1.16 (0.4-1.7) | 2.75 | 2.50E-03 | 9.20E-03 |
| tricarballylate | Energy | TCA Cycle | 0.69 (0.47-2.21) | 1.43 (0.81-7.84) | 2.64 | 0.020 | 0.060 |
| glucose 6-phosphate | Carbohydrate | Glycolysis, Gluconeogenesis, and Pyruvate Metabolism | 0.54 (0.38-0.91) | 0.61 (0.38-7.14) | 2.61 | 0.010 | 0.040 |
| 1-carboxyethylvaline | Amino Acid | Leucine, Isoleucine and Valine Metabolism | 0.48 (0.15-0.87) | 1.13 (0.47-1.72) | 2.59 | 5.50E-03 | 0.020 |
| trimethylamine n-oxide | Lipid | Phospholipid Metabolism | 0.3 (0.11-0.64) | 1.14 (0.39-2.3) | 2.48 | 0.020 | 0.060 |
| n-acetylasparagine | Amino Acid | Alanine and Aspartate Metabolism | 0.18 (0.18-0.25) | 0.63 (0.18-2.36) | 2.46 | 0.020 | 0.050 |
| 3-hydroxybutyrate (bhba) | Lipid | Ketone Bodies | 0.3 (0.3-0.58) | 0.97 (0.68-1.37) | 2.42 | 1.90E-04 | 9.80E-04 |
| 6-phosphogluconate | Carbohydrate | Pentose Phosphate Pathway | 0.28 (0.28-0.59) | 0.89 (0.28-2.48) | 2.39 | 2.10E-03 | 8.00E-03 |
| n-acetylvaline | Amino Acid | Leucine, Isoleucine and Valine Metabolism | 0.33 (0.31-0.63) | 1.2 (0.34-2.06) | 2.19 | 2.90E-03 | 0.010 |
| succinimide | Xenobiotics | Chemical | 0.26 (0.26-0.57) | 0.95 (0.49-1.56) | 2.18 | 1.50E-03 | 6.30E-03 |
| choline | Lipid | Phospholipid Metabolism | 0.83 (0.59-1.2) | 1.51 (0.89-2.93) | 2.16 | 0.050 | 0.110 |
| 2-hydroxyadipate | Lipid | Fatty Acid, Dicarboxylate | 0.76 (0.41-1.1) | 1.21 (0.66-2.96) | 2.14 | 6.80E-03 | 0.020 |
| kynurenate | Amino Acid | Tryptophan Metabolism | 0.27 (0.27-0.7) | 0.85 (0.42-3.55) | 2.13 | 0.020 | 0.050 |
| maltose | Carbohydrate | Glycogen Metabolism | 0.75 (0.3-1.14) | 1.94 (0.72-2.48) | 2.1 | 0.030 | 0.070 |
| n-acetylglycine | Amino Acid | Glycine, Serine and Threonine Metabolism | 0.66 (0.35-1.03) | 1.36 (0.79-2.18) | 2.08 | 1.50E-03 | 6.10E-03 |
| n-acetylglucosamine/n-acetylgalactosamine | Carbohydrate | Aminosugar Metabolism | 0.26 (0.26-0.72) | 0.91 (0.26-1.92) | 2.07 | 0.010 | 0.030 |
| ribose | Carbohydrate | Pentose Metabolism | 0.68 (0.2-0.96) | 1.29 (0.81-2.21) | 2.05 | 0.020 | 0.060 |
| 4-imidazoleacetate | Amino Acid | Histidine Metabolism | 0.21 (0.21-0.21) | 0.6 (0.21-1.29) | 2.02 | 0.010 | 0.030 |
| glutarate (c5-dc) | Lipid | Fatty Acid, Dicarboxylate | 0.69 (0.56-0.91) | 1.62 (0.97-2.03) | 2.02 | 5.30E-05 | 3.50E-04 |
| glycerophosphoinositol* | Lipid | Phospholipid Metabolism | 0.38 (0.36-0.77) | 1.04 (0.6-1.59) | 1.97 | 7.40E-03 | 0.020 |
| 2-phosphoglycerate | Carbohydrate | Glycolysis, Gluconeogenesis, and Pyruvate Metabolism | 0.62 (0.23-1.06) | 1.21 (0.74-1.95) | 1.95 | 0.050 | 0.110 |
| 3-hydroxyadipate* | Lipid | Fatty Acid, Dicarboxylate | 0.86 (0.32-0.98) | 1.34 (0.93-1.7) | 1.73 | 0.020 | 0.060 |
| methylmalonate (mma) | Lipid | Fatty Acid Metabolism (also BCAA Metabolism) | 0.79 (0.59-1.15) | 1.56 (0.64-2.98) | 1.66 | 0.050 | 0.110 |
| ceramide (d18:1/20:0, d16:1/22:0, d20:1/18:0)* | Lipid | Ceramides | 0.3 (0.3-0.74) | 0.71 (0.3-1.29) | 1.66 | 0.040 | 0.100 |
| 3-hydroxyisobutyrate | Amino Acid | Leucine, Isoleucine and Valine Metabolism | 0.3 (0.3-0.62) | 0.77 (0.3-1.16) | 1.58 | 0.030 | 0.060 |
| erucate (22:1n9) | Lipid | Long Chain Fatty Acid | 0.93 (0.81-1.08) | 1.1 (0.91-1.42) | 1.58 | 0.020 | 0.050 |
| mannose | Carbohydrate | Fructose, Mannose and Galactose Metabolism | 0.48 (0.45-1) | 0.92 (0.6-1.53) | 1.55 | 0.020 | 0.040 |
| arabitol/xylitol | Carbohydrate | Pentose Metabolism | 0.85 (0.74-1.16) | 1.35 (0.99-2.33) | 1.51 | 8.60E-03 | 0.03 |
| octanoylcarnitine (c8) | Lipid | Fatty Acid Metabolism(Acyl Carnitine) | 0.78 (0.45-1.22) | 0.45 (0.45-0.46) | -1.59 | 1.30E-03 | 5.60E-03 |
| gluconate | Xenobiotics | Food Component/Plant | 1.45 (0.94-1.79) | 0.82 (0.61-1.12) | -1.67 | 2.50E-03 | 9.10E-03 |
| tartarate | Xenobiotics | Food Component/Plant | 1.52 (0.81-3.23) | 0.68 (0.53-1.36) | -1.85 | 9.00E-03 | 0.03 |
| 2-methylbutyrylcarnitine (c5) | Amino Acid | Leucine, Isoleucine and Valine Metabolism | 0.83 (0.55-1.38) | 0.42 (0.42-0.44) | -1.86 | 8.00E-04 | 3.70E-03 |
| methionine | Amino Acid | Methionine, Cysteine, SAM and Taurine Metabolism | 1.87 (0.98-2.75) | 0.72 (0.64-1.16) | -1.95 | 1.60E-04 | 8.70E-04 |
| isovalerylcarnitine (c5) | Amino Acid | Leucine, Isoleucine and Valine Metabolism | 0.97 (0.42-1.54) | 0.4 (0.4-0.44) | -1.97 | 9.60E-04 | 4.40E-03 |
| n-acetylmethionine sulfoxide | Amino Acid | Methionine, Cysteine, SAM and Taurine Metabolism | 1.07 (0.81-2.05) | 0.4 (0.26-1.09) | -1.98 | 0.020 | 0.050 |
| propionylcarnitine (c3) | Lipid | Fatty Acid Metabolism (also BCAA Metabolism) | 1.08 (0.37-1.76) | 0.33 (0.23-0.82) | -1.99 | 0.020 | 0.060 |
| cysteinylglycine | Amino Acid | Glutathione Metabolism | 1.01 (0.4-1.5) | 0.35 (0.15-1) | -2 | 0.040 | 0.090 |
| 1-palmitoyl-2-oleoyl-gpc (16:0/18:1) | Lipid | Phosphatidylcholine (PC) | 1.19 (0.88-1.73) | 0.43 (0.18-1.23) | -2.01 | 0.040 | 0.090 |
| lactate | Carbohydrate | Glycolysis, Gluconeogenesis, and Pyruvate Metabolism | 1.68 (0.9-2.41) | 0.79 (0.3-1.08) | -2.05 | 0.040 | 0.100 |
| isobutyrylcarnitine (c4) | Amino Acid | Leucine, Isoleucine and Valine Metabolism | 0.61 (0.45-1.36) | 0.25 (0.25-0.26) | -2.2 | 2.00E-03 | 7.60E-03 |
| 1-oleoyl-2-linoleoyl-gpc (18:1/18:2)* | Lipid | Phosphatidylcholine (PC) | 1.01 (0.64-1.27) | 0.17 (0.17-0.87) | -2.29 | 7.10E-03 | 0.020 |
| leucylglutamine* | Peptide | Dipeptide | 0.99 (0.36-2.01) | 0.28 (0.28-0.43) | -2.33 | 1.90E-03 | 7.30E-03 |
| 3-hydroxyadipate* | Lipid | Fatty Acid, Dicarboxylate | 1.9 (0.86-3.24) | 0.72 (0.27-1.09) | -2.43 | 0.050 | 0.110 |
| 1-stearoyl-2-oleoyl-gpc (18:0/18:1) | Lipid | Phosphatidylcholine (PC) | 1.25 (0.79-1.99) | 0.3 (0.14-0.74) | -2.48 | 0.010 | 0.030 |
| acetylcarnitine (c2) | Lipid | Fatty Acid Metabolism(Acyl Carnitine) | 1.45 (0.82-1.99) | 0.47 (0.21-1.45) | -2.53 | 5.00E-03 | 0.020 |
| fructose 1,6-diphosphate/glucose 1,6-diphosphate/myo-inositol diphosphates | Carbohydrate | Glycolysis, Gluconeogenesis, and Pyruvate Metabolism | 1.13 (0.14-4.39) | 0.44 (0.1-0.8) | -2.67 | 0.040 | 0.090 |
| 1-palmitoyl-2-palmitoleoyl-gpc (16:0/16:1)* | Lipid | Phosphatidylcholine (PC) | 1 (0.66-1.57) | 0.17 (0.17-0.51) | -2.67 | 2.40E-03 | 8.80E-03 |
| serine | Amino Acid | Glycine, Serine and Threonine Metabolism | 2.59 (0.95-3.5) | 0.54 (0.4-1.07) | -2.73 | 8.00E-03 | 0.030 |
| histidine | Amino Acid | Histidine Metabolism | 1.78 (0.68-3.51) | 0.44 (0.26-1.37) | -2.78 | 0.010 | 0.040 |
| glycylleucine | Peptide | Dipeptide | 0.98 (0.33-1.59) | 0.24 (0.24-0.24) | -2.78 | 1.20E-05 | 9.70E-05 |
| ribulose/xylulose | Carbohydrate | Pentose Metabolism | 1.27 (0.47-3.3) | 0.24 (0.2-0.55) | -2.87 | 1.80E-03 | 7.20E-03 |
| isoleucine | Amino Acid | Leucine, Isoleucine and Valine Metabolism | 2.17 (1.02-3.33) | 0.55 (0.22-1.21) | -2.91 | 7.70E-03 | 0.020 |
| glycerophosphorylcholine (gpc) | Lipid | Phospholipid Metabolism | 1.02 (0.49-1.27) | 0.29 (0.03-2.07) | -2.96 | 0.040 | 0.100 |
| indolelactate | Amino Acid | Tryptophan Metabolism | 1.23 (0.7-2.57) | 0.25 (0.11-1.18) | -3.06 | 0.020 | 0.040 |
| threonylphenylalanine | Peptide | Dipeptide | 0.8 (0.27-1.26) | 0.18 (0.18-0.18) | -3.12 | 1.40E-05 | 1.00E-04 |
| glycerophosphoethanolamine | Lipid | Phospholipid Metabolism | 1.01 (0.41-1.55) | 0.07 (0.07-0.68) | -3.2 | 6.10E-03 | 0.02 |
| glycerophosphoglycerol | Lipid | Glycerolipid Metabolism | 3.24 (0.96-4.35) | 0.53 (0.25-1.01) | -3.23 | 3.90E-03 | 0.01 |
| stearoylcarnitine (c18) | Lipid | Fatty Acid Metabolism(Acyl Carnitine) | 1.07 (0.45-1.46) | 0.23 (0.23-0.25) | -3.25 | 3.60E-06 | 3.60E-05 |
| cysteine | Amino Acid | Methionine, Cysteine, SAM and Taurine Metabolism | 1.39 (0.41-3.4) | 0.39 (0.1-0.8) | -3.26 | 7.00E-03 | 0.02 |
| n-acetylmethionine | Amino Acid | Methionine, Cysteine, SAM and Taurine Metabolism | 1.04 (0.5-2.02) | 0.21 (0.02-0.97) | -3.48 | 0.030 | 0.080 |
| methionine sulfoxide | Amino Acid | Methionine, Cysteine, SAM and Taurine Metabolism | 1.88 (1.17-3.43) | 0.52 (0.19-0.97) | -3.53 | 1.30E-03 | 5.60E-03 |
| nicotinamide adenine dinucleotide (nad+) | Cofactors and Vitamins | Nicotinate and Nicotinamide Metabolism | 0.91 (0.43-1.57) | 0.1 (0.1-0.48) | -3.55 | 4.80E-04 | 2.30E-03 |
| tryptophan | Amino Acid | Tryptophan Metabolism | 2.55 (1.2-3.69) | 0.46 (0.19-0.79) | -3.78 | 4.70E-03 | 0.020 |
| leucylglycine | Peptide | Dipeptide | 0.95 (0.31-1.79) | 0.12 (0.12-0.19) | -3.81 | 1.60E-04 | 8.70E-04 |
| butyrylcarnitinec4 | butyrylcarnitine (C4) | Lipid | 1.23 (0.63-1.9) | 0.13 (0.13-0.61) | -3.82 | 5.10E-05 | 3.40E-04 |
| lysine | Amino Acid | Lysine Metabolism | 3.61 (1.35-4.89) | 0.68 (0.39-0.94) | -3.86 | 1.60E-04 | 8.70E-04 |
| leucine | Amino Acid | Leucine, Isoleucine and Valine Metabolism | 3.86 (1.61-5.56) | 0.79 (0.36-0.95) | -4.06 | 2.10E-04 | 1.10E-03 |
| hexanoylcarnitine (C6) | Lipid | Fatty Acid Metabolism(Acyl Carnitine) | 0.84 (0.13-1.07) | 0.13 (0.13-0.13) | -4.28 | 3.90E-07 | 5.20E-06 |
| thioproline | Xenobiotics | Chemical | 3.15 (1.07-4.64) | 0.47 (0.16-0.84) | -4.4 | 1.60E-03 | 6.40E-03 |
| sedoheptulose | Carbohydrate | Pentose Metabolism | 1.19 (0.86-2) | 0.15 (0.15-0.44) | -4.56 | 3.50E-07 | 5.20E-06 |
| isoleucylglycine | Peptide | Dipeptide | 0.92 (0.52-1.74) | 0.07 (0.07-0.23) | -4.76 | 4.30E-04 | 2.10E-03 |
| uridine | Nucleotide | Pyrimidine Metabolism, Uracil containing | 1.92 (0.88-3.35) | 0.44 (0.06-0.75) | -4.78 | 2.30E-04 | 1.20E-03 |
| cytosine | Nucleotide | Pyrimidine Metabolism, Cytidine containing | 2.27 (0.81-3.36) | 0.34 (0.04-0.82) | -4.81 | 1.70E-03 | 6.70E-03 |
| S-methylmethionine | Amino Acid | Methionine, Cysteine, SAM and Taurine Metabolism | 1.86 (0.56-2.91) | 0.14 (0.13-0.45) | -5 | 1.40E-05 | 1.10E-04 |
| lysylleucine | Peptide | Dipeptide | 0.79 (0.35-1.24) | 0.08 (0.08-0.08) | -5.03 | 1.10E-05 | 9.60E-05 |
| inosine | Nucleotide | Purine Metabolism, (Hypo)Xanthine/Inosine containing | 1 (0.5-6.13) | 0.09 (0.09-0.43) | -5.13 | 2.90E-03 | 0.01 |
| ornithine | Amino Acid | Urea cycle; Arginine and Proline Metabolism | 3.78 (1.36-8.02) | 0.53 (0.3-0.99) | -5.66 | 6.30E-05 | 4.10E-04 |
| phenylalanine | Amino Acid | Phenylalanine Metabolism | 3.98 (2-5.73) | 0.38 (0.15-0.91) | -7.34 | 1.30E-06 | 1.50E-05 |
| alanylleucine | Peptide | Dipeptide | 0.91 (0.33-1.55) | 0.06 (0.06-0.06) | -7.87 | 5.90E-07 | 7.10E-06 |
| valylleucine | Peptide | Dipeptide | 1.88 (0.88-2.63) | 0.07 (0.07-0.19) | -8.19 | 1.20E-05 | 9.60E-05 |
| valylglycine | Peptide | Dipeptide | 1 (0.43-1.98) | 0.06 (0.06-0.06) | -8.24 | 2.60E-06 | 2.70E-05 |
| phenylalanylglycine | Peptide | Dipeptide | 1.11 (0.35-2.03) | 0.05 (0.05-0.05) | -10 | 2.40E-07 | 3.90E-06 |
| hippurate | Xenobiotics | Benzoate Metabolism | 2.85 (0.28-7.86) | 0.06 (0.02-0.94) | -11.31 | 1.20E-03 | 5.40E-03 |
| valylglutamine | Peptide | Dipeptide | 1.03 (0.46-1.81) | 0.02 (0.02-0.08) | -12.33 | 5.70E-06 | 5.20E-05 |
| tyrosine | Amino Acid | Tyrosine Metabolism | 2.73 (1.37-4.92) | 0.07 (0.05-0.27) | -15.82 | 1.95E-08 | 4.20E-07 |
| tyrosylglycine | Peptide | Dipeptide | 1.18 (0.38-2.56) | 0.03 (0.03-0.03) | -16.46 | 7.67E-08 | 1.40E-06 |
| arginine | Amino Acid | Urea cycle; Arginine and Proline Metabolism | 8.91 (1.43-32.67) | 0.33 (0.11-0.61) | -20.89 | 3.70E-07 | 5.20E-06 |
| asparagine | Amino Acid | Alanine and Aspartate Metabolism | 2.5 (0.89-3.25) | 0.01 (0.01-0.04) | -63.21 | 5.30E-09 | 1.50E-07 |

| **Supplemental Table 2. Metabolic profile by birth outcome (n=40)** | | | | | | | |  |
| --- | --- | --- | --- | --- | --- | --- | --- | --- |
| **Metabolite** | **Superpathway** | **Subpathway** | **sPTB Median (IQR) (n=20)** | **Term Median (IQR) (n=20)** | **Fold Change** | **P-value** | **qvalue** | |
| maltotriose | Carbohydrate | Glycogen Metabolism | 1.18 (0.67-2.37) | 0.87 (0.16-1.94) | 2.78 | 0.042 | 0.98 | |
| glucose 6-phosphate | Carbohydrate | Glycolysis, Gluconeogenesis, and Pyruvate Metabolism | 0.61 (0.4-7.14) | 0.48 (0.38-0.91) | 2.36 | 0.027 | 0.98 | |
| adenosine | Nucleotide | Purine Metabolism, Adenine containing | 0.94 (0.47-2.18) | 0.41 (0.41-0.8) | 1.84 | 0.026 | 0.98 | |
| tartarate | Xenobiotics | Food Component/Plant | 1.25 (0.78-2.87) | 0.72 (0.59-1.53) | 1.61 | 0.045 | 0.98 | |
| maleate | Lipid | Fatty Acid, Dicarboxylate | 0.68 (0.66-1) | 1.52 (0.88-1.86) | -1.59 | 0.0023 | 0.71 | |

| **Supplemental Table 3. Metabolic profile among women with Community State Type I stratified by birth outcome (n=20)** | | | | | | | | |
| --- | --- | --- | --- | --- | --- | --- | --- | --- |
| **Metabolite** | **Subpathway** | **Superpathway** | **Term Median (IQR) (n=10)** | **sPTB Median (IQR) (n=10)** | **Fold Change** | **p-value** | **q-value** |  |
| inosine | Nucleotide | Purine Metabolism, (Hypo)Xanthine/Inosine containing | 0.5 (0.09-2.16) | 1.8 (0.91-6.43) | 4.41 | 0.044 | 0.99 |  |
| adenosine | Nucleotide | Purine Metabolism, Adenine containing | 0.47 (0.41-0.99) | 1.54 (0.88-4.57) | 2.49 | 0.044 | 0.99 |  |
| tartarate | Xenobiotics | Food Component/Plant | 0.91 (0.72-1.61) | 2.87 (1.14-4.23) | 2.19 | 0.013 | 0.99 |  |
| triethanolamine | Xenobiotics | Chemical | 0.61 (0.45-0.9) | 1.07 (0.81-1.48) | 1.86 | 0.011 | 0.99 |  |
| glutarate (C5-DC) | Lipid | Fatty Acid, Dicarboxylate | 0.91 (0.61-1.4) | 0.62 (0.54-0.72) | -1.52 | 0.017 | 0.99 |  |
| ceramide (d18:1/17:0, d17:1/18:0)* | Lipid | Ceramides | 0.67 (0.36-0.92) | 0.36 (0.36-0.36) | -1.56 | 0.036 | 0.99 |  |
| thymine | Nucleotide | Pyrimidine Metabolism, Thymine containing | 0.57 (0.4-0.79) | 0.25 (0.25-0.47) | -1.57 | 0.031 | 0.99 |  |
| nervonate (24:1n9)* | Lipid | Long Chain Fatty Acid | 0.76 (0.5-1.21) | 0.37 (0.37-0.64) | -1.58 | 0.045 | 0.99 |  |
| maleate | Lipid | Fatty Acid, Dicarboxylate | 1.3 (0.64-2.15) | 0.68 (0.67-0.83) | -1.62 | 0.046 | 0.99 |  |
| phosphoenolpyruvate (PEP) | Carbohydrate | Glycolysis, Gluconeogenesis, and Pyruvate Metabolism | 0.96 (0.83-1.34) | 0.35 (0.15-0.55) | -2.72 | 0.018 | 0.99 |  |

| **Supplemental Table 4. Metabolic profile among women with Community State Type IV stratified by birth outcome (n=20)** | | | | | | | |
| --- | --- | --- | --- | --- | --- | --- | --- |
| **Metabolite** | **Subpathway** | **Superpathway** | **Term Median (IQR) (n=10)** | **sPTB Median (IQR) (n=10)** | **Fold Change** | **p-value** | **q-value** |
| glucose 6-phosphate | Carbohydrate | Glycolysis, Gluconeogenesis, and Pyruvate Metabolism | 7.14 (0.66-14.63) | 0.38 (0.38-0.49) | 6.69 | 0.0037 | 0.57 |
| 3-methylhistidine | Amino Acid | Histidine Metabolism | 1.38 (0.91-3.16) | 0.12 (0.05-0.73) | 6.37 | 0.0067 | 0.67 |
| 2-keto-3-deoxy-gluconate | Xenobiotics | Food Component/Plant | 1.18 (0.5-2.11) | 0.34 (0.23-0.41) | 3.15 | 0.014 | 0.70 |
| kynurenate | Amino Acid | Tryptophan Metabolism | 2.47 (0.98-4.36) | 0.52 (0.27-0.72) | 3.03 | 0.021 | 0.700 |
| phenyllactate (PLA) | Amino Acid | Phenylalanine Metabolism | 1.28 (0.46-2.05) | 0.57 (0.29-0.83) | 2.77 | 0.047 | 0.830 |
| 6-oxopiperidine-2-carboxylate | Amino Acid | Lysine Metabolism | 0.78 (0.44-2.14) | 0.22 (0.2-0.31) | 2.71 | 0.012 | 0.700 |
| pyruvate | Carbohydrate | Glycolysis, Gluconeogenesis, and Pyruvate Metabolism | 1.49 (1.12-2.43) | 0.81 (0.18-1.1) | 2.69 | 0.027 | 0.810 |
| ribulose/xylulose | Carbohydrate | Pentose Metabolism | 0.54 (0.29-1.47) | 0.2 (0.2-0.2) | 2.66 | 0.0038 | 0.570 |
| 2-hydroxyadipate | Lipid | Fatty Acid, Dicarboxylate | 2.56 (0.97-4.11) | 0.97 (0.53-1.34) | 2.48 | 0.037 | 0.830 |
| ribulonate/xylulonate* | Carbohydrate | Pentose Metabolism | 2.14 (1.32-2.99) | 0.82 (0.72-1.46) | 2.14 | 0.019 | 0.700 |
| maleate | Lipid | Fatty Acid, Dicarboxylate | 0.78 (0.66-1.3) | 1.6 (1.14-1.69) | -1.57 | 0.021 | 0.700 |
| spermine | Amino Acid | Polyamine Metabolism | 0.04 (0.04-0.04) | 0.93 (0.04-1.91) | -6.91 | 0.030 | 0.810 |
| hippurate | Xenobiotics | Benzoate Metabolism | 0.02 (0.02-0.06) | 0.71 (0.06-2.8) | -9.14 | 0.019 | 0.700 |

SUPPPLEMENTAL FIGURE


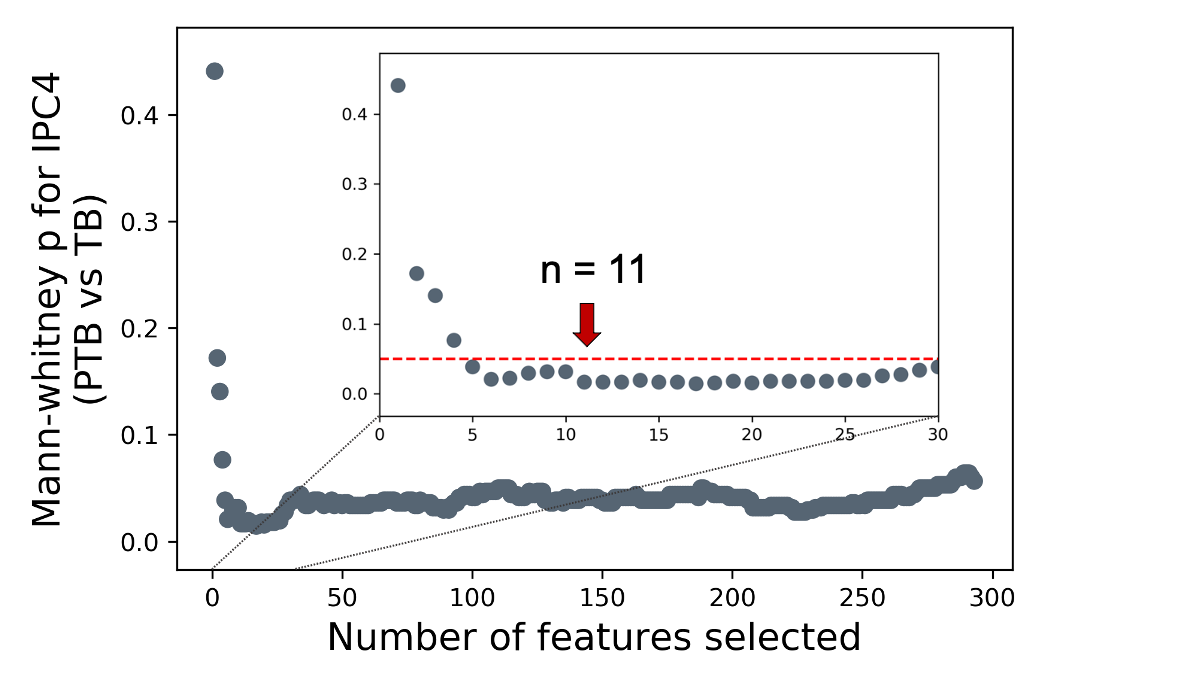


**Fig. S1 Scatter plot of the number of features being selected using sparse IPCA (sIPCA) and corresponding *P* values of Mann-Whitney U tests on the difference between IPC4 components of spontaneous preterm birth and term birth.** A zoomed-in view for a selection of 1-30 features is presented. Red dashed line shows the *P* level of 0.05. P value is the lowest when 11 features are selected.
